# Glycation of paraoxonase 1 by high glucose instigates endoplasmic reticulum stress to induce endothelial dysfunction *in vivo*

**DOI:** 10.1038/srep45827

**Published:** 2017-04-04

**Authors:** Wei Yu, Xiaoli Liu, Liru Feng, Hui Yang, Weiye Yu, Tiejian Feng, Shuangxi Wang, Jun Wang, Ning Liu

**Affiliations:** 1 Central Laboratory, Second Hospital, Jilin University, Changchun 130041, China; 2 Shenzhen Center for Chronic Disease Control, Shenzhen 518020, China; 3 Department of Pharmacology, College of Pharmacy, Xinxiang Medical University, Xinxiang, 453003, China

## Abstract

High-density lipoprotein (HDL) modulates low-density lipoprotein and cell membrane oxidation through the action of paraoxonase-1 (PON1). Endoplasmic reticulum (ER) stress has been linked to a wide range of human pathologies including diabetes, obesity, and atherosclerosis. Previous studies have reported that PON1 is glycated in diabetes. The aim of this study is to investigate whether and how PON1 glycation contributes to endothelial dysfunction in diabetes. ER stress markers were monitored by western blot. Endothelial function was determined by organ bath. Incubation of recombinant PON1 proteins with high glucose increased PON1 glycation and reduced PON1 activity. Exposure of HUVECs to glycated PON1 induced prolonged ER stress and reduced SERCA activity, which were abolished by tempol, apocynin, BAPTA, and p67 and p22 siRNAs. Chronic administration of amino guanidine or 4-PBA prevented endothelial dysfunction in STZ-injected rats. Importantly, injection of glycated PON1 but not native PON1 induced aberrant ER stress and endothelial dysfunction in rats, which were attenuated by tempol, BAPTA, and 4-PBA. In conclusion, glycation of PON1 by hyperglycemia induces endothelial dysfunction through ER stress. In perspectives, PON1 glycation is a novel risk factor of hyperglycemia-induced endothelial dysfunction. Therefore, inhibition of oxidative stress, chelating intracellular Ca^2+^, and ER chaperone would be considered to reduce vascular complications in diabetes.

Diabetes mellitus is usually associated with the development of atherosclerosis and nephropathy, which is characterized by endothelial dysfunction^1^,^2^. Advanced glycation end-products (AGEs) are a heterogeneous group of products which protein and lipids are covalently bound to sugar residues under hyperglycemic and oxidative stress situations, which is proposed to play a major role in the pathogenesis of diabetic complications^3^.

High-density lipoprotein (HDL) associated paraoxonase-1 (PON1) is primarily responsible for the anti-oxidative properties of HDL in retarding the oxidation of low-density lipoprotein (LDL) and cell membranes^4^,^5^,^6^. By modulating the oxidation of LDL, PON1 abolishes the ox-LDL-stimulated induction of monocyte-chemotactic protein-1 (MCP1) produced by endothelial cells, thereby preventing monocyte/endothelial cell interaction in one of the earliest processes of atherosclerosis^7^,^8^. PON1 is low in subjects with diabetes, leading to dysfunctional HDL with impaired antioxidant capacity^9^,^10^,^11^. In diabetes, there is an inverse relationship between PON1 activity and circulating oxidized LDL levels, indicative of the major role of PON1 in retarding LDL oxidation^12^,^13^. Glycation of paraoxonase-1 inhibits its activity and impairs the ability of HDL to metabolize membrane lipid hydroperoxides^14^. It has been reported that dysfunction of PON is related to vascular oxidative stress^15^ and vascular damage^5^,^16^,^17^. However, the mechanism needs to be defined.

The normal endoplasmic reticulum (ER) is the principal site of protein synthesis, folding, and maturation. ER stress has been linked to a wide range of human pathologies including diabetes, obesity, atherosclerosis, cancer, neurodegenerative disorders, and inflammatory conditions^18^,^19^. ER stress may be triggered by hyperglycemia, oxidative stress, Ca^2+^ overload, ischemia, and hypoxia. In normal condition, unfolded or misfolded proteins in ER are sent to the cytoplasm by a “retro-translocation mechanism” to be degraded by the ubiquitin proteasome system^20^. However, ER stress causes the accumulation of unfolded and misfolded proteins, leading to an “unfolded protein response (UPR)”, resulting in cellular dysfunctions^21^. Previous studies have shown that glycation of LDL triggered ER Stress and induced endothelial dysfunction^22^,^23^.

Based on the literature evidence, we hypothesized that PON1 glycation may promote endothelial dysfunction via ER stress. In this study, we reported that recombinant PON1 protein was glycated by high glucose *in vitro*. Glycated PON1 (Gly-PON1) instigated ER stress via the oxidation and inhibition of sarcoplasmic/endoplasmic reticulum Ca^2+^ ATPase (SERCA) in endothelial cells and induced endothelial dysfunction in rats. In perspectives, PON1 glycation is a risk factor of endothelial dysfunction in diabetes.

## Materials and Methods

### Materials

Antibodies against phospho-eukaryotic translation initiation factor 2α (eIF2α), and 3-nitrotryosine (3-NT) were obtained from Cell Signaling Biotechnology (Danvers, MA). The antibodies against phospho-PKR (protein kinase R)-like ER kinase (PERK), CHOP, ATF6, BIP, SERCA, scrambled small interfering RNA (siRNA), and the specific siRNA for p67 and p22 were obtained from Santa Cruz Biotechnology Inc. (Santa Cruz, CA). Amino guanidine (AG), streptozotocin (STZ), tempol, 1,2-bis (2-aminophenoxy) ethane-N4-tetraacetic acid (BAPTA), 4-phenyl butyric acid (4-PBA), tunicamycin, D-glucose, acetylcholine (ACh), sodium nitroprusside (SNP), phenylephrine (PE) and dihydroethidium (DHE) were purchased from Sigma-Aldrich Company or Caymen chemical Company. Fluo-4 NW kits were obtained from Invitrogen Inc. (Carlsbad, CA). All other chemicals, if not indicated, were purchased from Sigma-Aldrich (St. Louis, MO).

### Preparation of glycated of PON1

To prepare glycated PON1, recombinant PON1 protein (10 μg) from Abcam Company was incubated with 0.3 mmol/l EDTA at 37 °C, pH 7.4 for 3 days in freshly prepared D-glucose. For normal and glycated PON1, 1 mmol/l DTPA was also added and incubations were under nitrogen. Modification was terminated by repeat extensive dialysis as described above^24^. Highly glycated PON1 was generated by buffer exchange of native PON1 into PBS, pH 7.4, dilution to 3 mg/ml protein, and addition of CuCl_2_ to a final concentration of 10 μmol/l for 24 hours, under air at 37 °C. PON1 preparations were sterile filtered (0.22 μm), stored in the dark under nitrogen at 4 °C, and used within 1 month of preparation. The PON1 pools were tested for endotoxin contamination by the Limulus Amebocyte Lysate (Bio-Whittaker, Walkersville, MD) according to the manufacturer’s suggestion.

### Determination of PON1 activity

As described previously^14^, PON1 activity was measured by adding 20 μL of sample to Tris buffer (100 mmol/L, pH 8.0) containing 2 mmol/l CaCl_2_ and 1 mmol/L paraoxon (Sigma). PON1 activity was measured using phenylacetate as a substrate and the reaction mixture contained 750 μL of 0.1 mol/L Tris- HCl (pH 8.5), 1 mmol/L CaCl_2_, 125 μL of 12 mmol/L phenylacetate and 125 μL of diluted serum with water (1:10). Initial rates of hydrolysis were determined by following the increase of phenol concentration at 270 nm at 37 °C. Enzyme activities were expressed in international units per 1 liter of serum (U/L). An international unit is the amount of hydrolyzed substrate in mmol/minute.

### Cell cultures

Human umbilical vein endothelial cells (HUVECs) were grown in EBM (Clonetics Inc. Walkersville, MD) supplemented with 2% fetal bovine serum, penicillin (100 u/ml), and streptomycin (100 μg/ml). In all experiments, cells were between passages 3 and 8. All cells were incubated at 37 °C in a humidified atmosphere of 5% CO_2_ and 95% air. Cells were grown to 70–80% confluency before being treated with different agents.

### Transfection of siRNA into cells

Transient transfection of siRNA was carried out according to Santa Cruz’s protocol^25^. Briefly, the siRNAs were dissolved in siRNA buffer (20 mM KCl; 6 mM HEPES, pH 7.5; 0.2 mM MgCl_2_) to prepare a 10 μM stock solution. Cells grown in 6-well plates were transfected with siRNA in transfection medium containing liposomal transfection reagent (Lipofectamine RNAiMax, Invitrogen, Shanghai branch, China). For each transfection, 100 μl transfection medium containing 4 μl siRNA stock solution was gently mixed with 100 μl transfection medium containing 4 μl transfection reagent. After 30-min incubation at room temperature, siRNA-lipid complexes were added to the cells in 1.0 ml transfection medium, and cells were incubated with this mixture for 6 h at 37 °C. The transfection medium was then replaced with normal medium, and cells were cultured for 48 h.

### Western blotting

As described previously^26^, cells or aortic tissues were homogenized on ice in cell-lysis buffer (20 mM Tris-HCl, pH 7.5, 150 mM NaCl, 1 mM Na_2_EDTA, 1 mM EGTA, 1% Triton, 2.5 mM sodium pyrophosphate, 1 mM beta-glycerophosphate, 1 mM Na_3_VO_4_, 1 μg/ml leupeptin, and 1 mM PMSF). Cell was lysated with cell-lysis buffer. The protein content was assayed by BCA protein assay reagent (Pierce, USA). 20 μg proteins were loaded to SDS-PAGE and then transferred to membrane. Membrane was incubated with a 1:1000 dilution of primary antibody, followed by a 1:2000 dilution of horseradish peroxidase- conjugated secondary antibody. Protein bands were visualized by ECL (GE Healthcare). The intensity (area X density) of the individual band on western blots was measured by densitometry (model GS-700, Imaging Densitometer; Bio-Rad). The background was subtracted from the calculated area.

### Measurement of intracellular Ca^2+^ and SERCA activity

Intracellular Ca^2+^ concentration was measured using a Fluo-4 NW kits in accordance with manufacturer’s recommendations with the fluorophore excited at 485 nm and detection at 520 nm. Ca^2+^-uptake by SERCA was determined radiometrically using a rapid filtration technique as described previously^27^. Cells were incubated at 37 °C in 1.5 ml of buffer B (40 mM imidazole, pH 7.0, 100 mM KCl, 5 mM MgCl_2_, 5 mM NaN_3_, 5 mM potassium oxalate, 0.5 mM EGTA, 1 μM ruthenium red), 10 μCi Ca^2+^ and CaCl_2_, to yield the required final concentration of free Ca^2+^. Ca^2+^-uptake was initiated by addition of ATP, and terminated after 1, 3 and 5 sec by addition of 3 ml of ice-cold washing solution (20 mM HEPES, pH 7.4, 150 mM KCl, 1.4 mM MgCl_2_, and 2 mM KH_2_PO_4_), followed by filtration through a HAWP 0.45 μm Millipore filter in a Millipore filtration device. The initial rate of Ca^2+^-uptake was calculated by linear regression analysis.

### Biotinylated-iodoacetamide labeling of SERCA Cysteine-674

The method for biotinylated-iodoacetamide (b-IAM) labeling of the reactive thiol on cysteine-674 in SERCA followed those previously reported with modifications^28^. Briefly, cells were lysed in buffer A (Tris-HCL 50 mmol/L pH8.5, NaCl 150 mmol/L, MgCl_2_ 5 mmol/L, DETA-PAC 50 μmol/L, PMSF 2 mmol/L, Triton 0.5% and 100 μmol/L NEM) on ice for 50 min. Pretreatment with this low concentration of NEM was used to minimize incorporation of b-IAM label into protein that was partially denatured during cell lysis. The excess NEM was removed by gel filtration using Biospin 6 columns (Biorad). The cell lysate was then incubated with b-IAM (1 mmol/L) in buffer B (MES 50 mmol/L pH 6.5, NaCl 150 mmol/L, MgCl_2_ 5 mmol/L, DETA-PAC 50 μmol/L, PMSF 2 mmol/L and Triton X-100 1%) in the dark at 25 °C for 30 minutes. The labeling reaction was terminated by adding β-mercaptoethanol (β-ME) to a final concentration of 50 mmol/L. The excess reagent was removed by Biospin 6 columns. Finally, 500 μg cell lysate protein was incubated with 50 μL streptavidin-Sepharose beads overnight at 4 °C. The beads were rinsed 3 times using buffer C (Tris-HCl l25 mmol/L pH 7.4, NaCl 500 mmol/L, MgCl_2_ 5 mmol/L and 2% SDS), the b-IAM labeled proteins were released by Laemmli buffer with 5 mol/L urea and 5% β-ME at 55 °C for at least 30 min. Proteins were separated by SDS-PAGE, and SERCA was detected by immunoblot with anti-SERCA monoclonal antibody (Affinity Bioreagent, IID8 910, 1:2,000).

### Animal experimental protocols

Male Sprague-Dawley (SD) rats (8 ± 2 weeks old, 180 ± 20 g) were purchased from Hua-Fu-Kang Animal Company (Beijing, China). All rat*s* were housed individually in cages at a room temperature of 21 ± 1 °C with a 12-h light/dark cycle and given free access to food and water. This study was carried out in strict accordance with the recommendations in the Guide for the Care and Use of Laboratory Animals of the National Institutes of Health. The protocol was approved by the Committee on the Ethics of Animal Experiments of Jilin University.

For the first part of the animal study to generate diabetic model of rats, SD rats were administrated with amino guanidine (100 mg/kg per day) or 4-PBA (1 g/kg/day) for 2 weeks and during the whole experiments. Then rats were received injection of a low-dose STZ (50 mg/kg/day, 5 consecutive days, I.P.) to induce pancreatic islet cell destruction and persistent hyperglycemia recommended by the Animal Models of Diabetic Complications Consortium. Hyperglycemia was defined as a random blood glucose level of >300 mg/dl for >2 weeks after injection. Four weeks after STZ injection, the rats in each group were anesthetized with sodium pentabarbitone (30 mg/kg, I.P.) and exsanguinated. Aortas from rats were cut into rings and were mounted in organ chamber to detect vessel bioactivity. Blood was collected to measure serum levels of glycated PON1 and PON1 activity in rats.

For the second part of the animal study, SD rats were administrated with tempol (1 mM in the drinking water), 4-PBA (1 g/kg/day), and BAPTA (5 mg/kg per day) for 2 weeks followed by injection of glycated PON1 (1 mg/kg/day, 7 consecutive days) via tail vein. At the end of experiments, all rats were anesthetized with sodium pentabarbitone (30 mg/kg, I.P.) and exsanguinated. Aortas from rats were cut into rings and were mounted in organ chamber to detect vessel bioactivity. Arterial walls were stained with DHE to determine superoxide productions. Western blot was performed to measure the levels of ER stress markers.

### Measurement of glycated PON1

The concentration of glycated PON1 in serum was determined by boronate affinity chromatography^14^. Briefly, serum was diluted 1:20 with PBS containing 1% triton X100 to dissociate the HDL complex and subject to maminophenylboronate affinity chromatography. Glycated and non-glycated PON1 were then determined by our in-house ELISA. Briefly, standards, glycated and non-glycated PON1 samples were diluted 1:4500 in 0.05 M carbonate buffer pH 9.6, 100 μl added to duplicate wells of a 96-well plate and incubated for 16 hours at room temperature (22 °C). Wells were washed with PBS pH 7.4 containing 0.1% bovine serum albumin (PBS/BSA) and incubated with PBS/1% BSA for 1 h at room temperature. Following washing (X3) rabbit anti-human PON1 IgG diluted 1:6400 in PBS/1% BSA was added and incubated for 1 h at room temperature. Wells were washed (X2), anti-rabbit peroxidase conjugate (1:2500) added and incubated for 1 h at room temperature. Wells were then washed (X3) and tetramethylbenzidine substrate added. After 15 min at room temperature, 2 M sulphuric acid was added and the absorbance read at 450 nm. Recovery of PON1 in the two fractions was 96 ± 1.8% by reference to the original serum value.

### Detection of superoxide

As described previously^29^,^30^, to measure superoxide production in cultured cells or in the artery *in situ*, fresh frozen sections of aorta arch were isolated from rats, and were stained with 10 μM DHE for 30 min, rinsed, and observed by fluorescent microscopy. Results were quantified using BIOQUANT Image software.

### DHE-derived fluorescence assay of NADPH oxidase activity in the microplate reader

The NADPH oxidase activity was measured, as described previously^31^. Briefly, 20 μg protein was incubated with DHE (10 μM) and DNA (1.25 μg/ml) in PBS with the addition of NADPH (50 μM), in a final volume of 120 μl. Incubations were performed for 30 min at 37 °C in the dark. Fluorescence intensity was recorded in a microplate reader (excitation 490 nm and emission 590 nm).

### Measurement of tension development in aortic rings

*In vivo* or *ex vivo* organ chamber study was performed as we described previously^32^,^33^. Rats were sacrificed under anesthesia by intravenous injection with pentobarbital sodium (30 mg/kg). The descending aorta isolated by removing the adhering perivascular tissue carefully was cut into rings (3–4 mm in length). Aortic rings were suspended and mounted to organ chamber by using two stainless. The rings were placed in organ baths filled with Kreb’s buffer of the following compositions (in mM): NaCl, 118.3; KCl, 4.7; MgSO_4_, 0.6; KH_2_PO_4_, 1.2; CaCl_2_, 2.5; NaHCO_3_, 25.0; EDTA, 0.026; pH 7.4 at 37 °C and gassed with 95% O_2_ plus 5% CO_2_, under a tension of 2.0 g for 90-minute equilibration period. During this period, the Kreb’s solution was changed every 15 min. After the equilibration, aortic rings were challenged with 60 mM KCl. After washing and another 30 minutes equilibration period, contractile response was elicited by PE (1 μM). At the plateau of contraction, accumulative Ach or SNP was added into the organ bath to induce the vasorelaxation. The relaxation was calculated as a ratio of the Ach/SNP-induced vasodilation to PE-elicited vasoconstriction. The ratio at 1 was set as 100% of relaxation.

### Measurements of blood glucose, cholesterol, and triglyceride

The determinations of blood glucose, cholesterol, and triglyceride were assayed by using commercial kits as recommend by the manufacturer of Jian-Cheng Bioengineering Institute (Nanjing, China).

### Statistical Analysis

Values are reported as mean ± SEM with sample sizes indicated in each legend. Statistical comparisons of vasodilation were performed with repeated-measures ANOVA, and intergroup differences were tested with Bonferroni inequality. Other data were analyzed with a 1-way ANOVA followed by Bonferroni *post-hoc* analyses. All statistical analyses were performed using GraphPad Prism 4 analysis software (La Jolla, CA). A two-sided *P*-value* *<* *0.05 was considered significant.

## Results

### High glucose induces PON1 glycation and reduces PON1 activity

**Figure 1:**
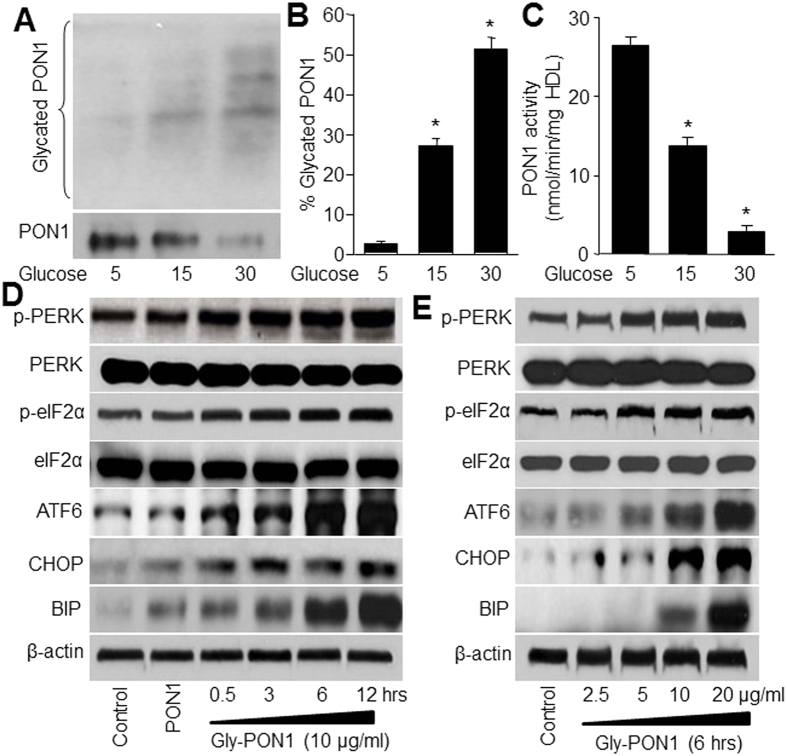
High glucose induces PON1 glycation and ER stress. (**A**–**C**) Recombinant PON1 protein (10 μg) was incubated with D-glucose (5, 15, 30 mM) for 7 days in reaction buffer. The level of glycated PON1 was determined by running and staining SDS-PAGE with coomassie brilliant blue in (**A**) and was calculated in (**B**). PON1 activity was measured in (**C**). N = 3 per group. *P < 0.05 *vs*. Control (5 mM D-glucose). (**D**) Cultured HUVECs were incubated with native or glycated PON1 (10 μg/ml) as indicated times (0.5–12 hours). (**E**) HUVECs were incubated with glycated PON1 as indicated concentrations (2.5, 5, 10, 20 μg/ml) for 6 hours. The levels of p-PERK, p-eIF2α, CHOP, ATF6 and BIP were assayed by western blot in total cell lysates from (**D**,**E**). The blot was a representative picture from 3 independent experiments and cropped from the full blot shown in Supplementary Figures S1, S2 and S3.

To test whether PON1 protein is glycated by high glucose, recombinant PON1 protein was incubated with D-glucose and SDS-PAGE analysis was performed to detect the modifications of PON1 protein by glucose. As shown in Fig. 1A and B, following treatment of 15–30 mM D-glucose for 7 days, PON1 protein was significantly glycated by high glucose. Further, incubation of PON1 protein with high glucose remarkably reduced PON1 activity (Fig. 1C). These data indicate that PON1 is glycated and inhibited by high glucose.

### Glycated PON1 increases ER stress in endothelial cells

It has been reported that highly glycated LDL induces ER stress to impair endothelial function^22^. Thus, we determined whether gly-PON1, similar to glycated LDL, triggers ER stress. To test this notion, confluent HUVECs were exposed to gly-PON1 at a series concentrations of 10 μg/ml, which is considered to be pathologically relevant to diabetes, for 30 min to 12 hours. HUVECs exposed to native PON1 (10 μg/ml) were used as controls. As depicted in Fig. 1D, gly-PON1 but not native PON1, markedly increased the detection of ER stress markers including p-PERK, p-eIF2α, CHOP, ATF6 and BIP, as early as 30 minutes after gly-PON1 exposure.

The effects of gly-PON1 appeared to be dose dependent. Low concentration of gly-PON1 (2.5 μg/ml) had no effects on ER stress markers including p-PERK, p-eIF2α, CHOP, ATF6 and BIP, while increased concentrations of gly-PON1 (5–20 μg/ml) markedly increased the detection of ER stress markers within 6-hour exposure (Fig. 1E).

### ER stress triggered by gly-PON1 is oxidative stress dependent

**Figure 2:**
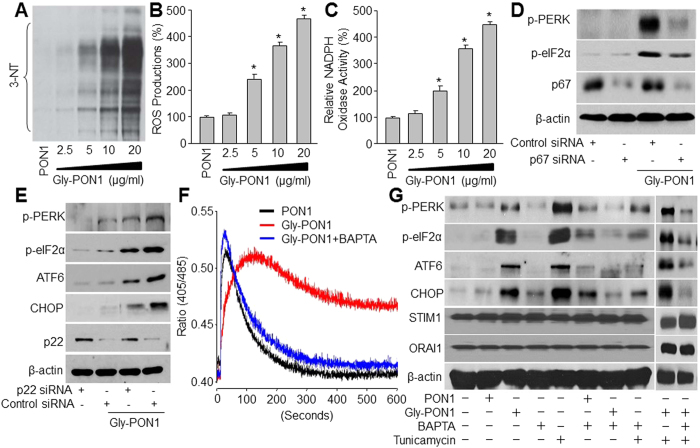
Glycated PON1 triggers oxidative stress to induce ER stress and intracellular Ca^2+^ overload in HUVECs. (**A**–**C**) HUVECs were incubated with glycated PON1 as indicated concentrations (2.5, 5, 10, 20 μg/ml) for 6 hours. The 3-NT level was assayed in total cell lysates by western blot in (**A**). NADPH oxidase activity in (**B**) and ROS production in (**C**) were also assessed. N = 3 per group. *P < 0.05 *vs*. Control (PON1 alone). (**D**,**E**) HUVECs were transfected with p67 siRNA in (**D**) or p22 siRNA in (**E**) for 48 hours followed by co-incubation with glycated PON1 (10 μg/ml, 6 hours). The levels of p-PERK, p-eIF2α, CHOP and ATF6 were assayed in total cell lysates by western blot. The blot was a representative picture from 3 independent experiments. (**F**) HUVECs were treated with 10 μg/ml gly-PON1 with or without BAPTA (0.5 mM) for 6 hours. Ratiometric measurement of intracellular Ca^2+^ was done. N = 3 per group. (**G**) HUVECs were treated with 10 μg/ml gly-PON1 and tunicamycin (10 μM) in presence or absence of BAPTA (0.5 mM) from 6 hours. The levels of p-PERK, p-eIF2α, CHOP, ATF6, STIM1 and ORAI1 were assayed in total cell lysates by western blot. These experiments were repeated for 3 times. The presented blots were cropped from the full blots shown in Supplementary Figures S4–8.

Oxidative stress is known to increase both intracellular Ca^2+^ and ER stress in endothelial cells^24^. Peroxynitrite is a potent oxidant formed by the combination of nitric oxide and superoxide. 3-NT positive proteins are considered a footprint of peroxynitrite in cultured cells^34^. We next first assayed whether gly-PON1 increased the levels of 3-NT. As depicted in Fig. 2A and B, exposure of HUVECs to gly-PON1 dose-dependently increased the levels of 3-NT^+^ proteins and ROS productions, implying that gly-PON1 triggers oxidative stress in endothelial cells.

NAD(P)H oxidase is a major source of superoxide endothelial cells^31^. Next we determined whether genetic inhibition of NAD(P)H oxidase attenuated ER stress caused by gly-PON1. As depicted in Fig. 2C, exposure of HUVECs to gly-PON1 dose-dependently increased the activity of NAD(P)H oxidase. Further, transfection of p67-specific or p22-sepecific siRNA significantly reduced the levels of p67 and p22, both essential components of NAD(P)H oxidases^31^, abrogated gly-PON1-induced ER stress (Fig. 2D and E). These data suggest that NAD(P)H oxidase-derived superoxide is required for gly-PON1-triggered ER stress.

### Chelation of intracellular Ca^2+^ abolishes gly-PON1-induced ER stress

We next determined whether the rise of intracellular Ca^2+^ concentration ([Ca^2+^]_i_) contributed to increased detection of ER stress. As shown in Fig. 2F and G, compared to PON1, gly-PON1 dramatically increased [Ca^2+^]_i_. However, pretreatment of HUVECs with Ca^2+^ chelator BAPTA (10 μmol/l) significantly inhibited gly-PON1-induced intracellular Ca^2+^ overload and increased expressions of ER stress markers, implying that a rise of [Ca^2+^]_i_ was critical for gly-PON1-enhanced ER stress. Interestingly, compared to PON1, gly-PON1 did not affect the protein levels of STIM1 and ORAI1, which were not altered by BAPTA and tunicamycin or plus, either. This indicates that SERCA oxidation plays a predominant role in gly-PON1-induced Ca^2+^ overload and ER stress.

The role of intracellular Ca^2+^ overload on ER stress was further confirmed by using tunicamycin, which has been reported to increase [Ca^2+^]_i_ and trigger ER stress^35^. As depicted in Fig. 2F, consistently, tunicamycin increased ER stress in HUVECs, which were abolished by chelating [Ca^2+^]_i_ with BAPTA. Moreover, BAPTA abolished ER stress in cells treated with gly-PON1 plus tunicamycin, further supporting the hypothesis.

### Gly-PON1 reduces SERCA activity along with increased detection of SERCA oxidation

**Figure 3:**
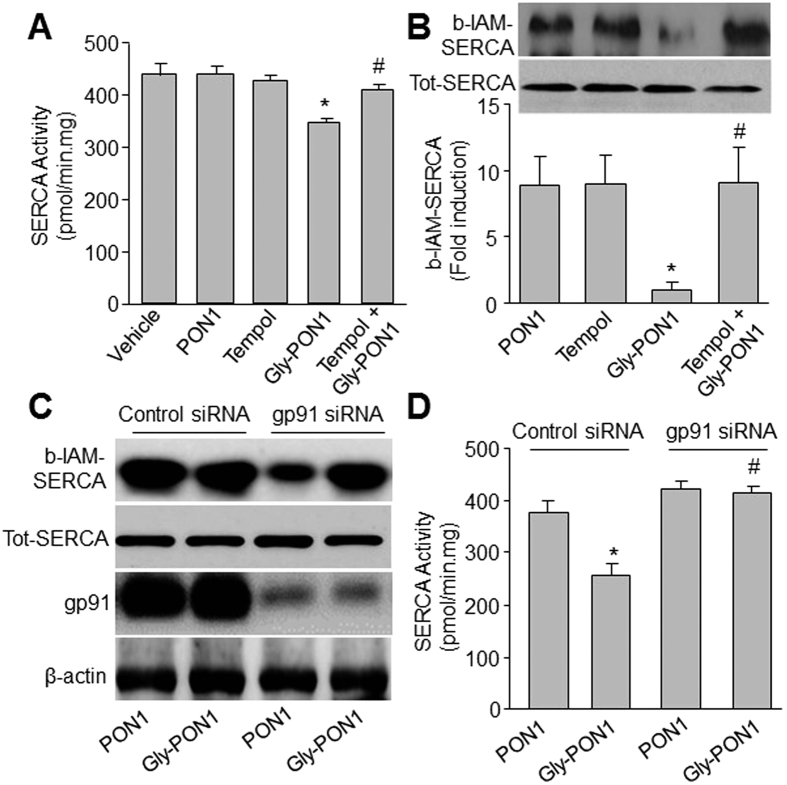
Glycated PON1 decreased SERCA activity by oxidation. (**A**,**B**) HUVECs were incubated with or without native PON1 (10 μg/ml), glycated PON1 (10 μg/ml), tempol (10 μM) and glycated PON1 plus tempol for 6 hours. SERCA activity was assayed in (**A**) by measuring Ca^2+^ uptake and release. SERCA oxidation in (**B**) was detected by using b-IAM labeling of SERCA cysteine 674. N = 3 per group. *P < 0.05 *vs*. Vehicle or PON1 alone. ^#^*P *<* *0.05 *vs*. Gly-PON1 alone. (**C**,**D**) HUVECs were transfected with gp91 siRNA for 48 hours followed by co-incubation with glycated PON1 (10 μg/ml, 6 hours). SERCA oxidation and gp91 protein were assessed in (**C**). SERCA activity in (**D**) was assayed in by measuring Ca^2+^ uptake and release. N = 3 per group. *P < 0.05 *vs*. PON1 alone. ^#^*P* < 0.05 *vs*. Gly-PON1 alone. The presented blots were cropped from the full blots shown in Supplementary Figures S9 and S10.

To explore how gly-PON1 exposure could trigger ER stress in endothelial cells, we determined whether gly-PON1 suppressed SERCA activity by oxidation. As depicted in Fig. 3A and B, gly-PON1 significantly inhibited SERCA activity and increased SERCA oxidation by detecting b-IAM-labeled SERCA cysteine 674, which is reported to be oxidized and suppressed by superoxide^28^ in HUVECs. Further, tempol, a superoxide scavenger^31^, prior to the addition of gly-PON1, significantly attenuated gly-PON1-reduced SERCA activity and SERCA oxidation.

We also tested the effects of NAD(P)H oxidase on SERCA activity and oxidation. As shown in Fig. 3C and D, inhibition of NAD(P)H oxidase by gp91 siRNA significantly inhibited gly-PON1-induced reduction of SERCA activity and SERCA oxidation, suggesting that gly-PON1 attenuates SERCA activity via NAD(P)H oxidase.

### AGEs inhibitor AG prevents endothelial dysfunction in STZ-injected rats

**Figure 4:**
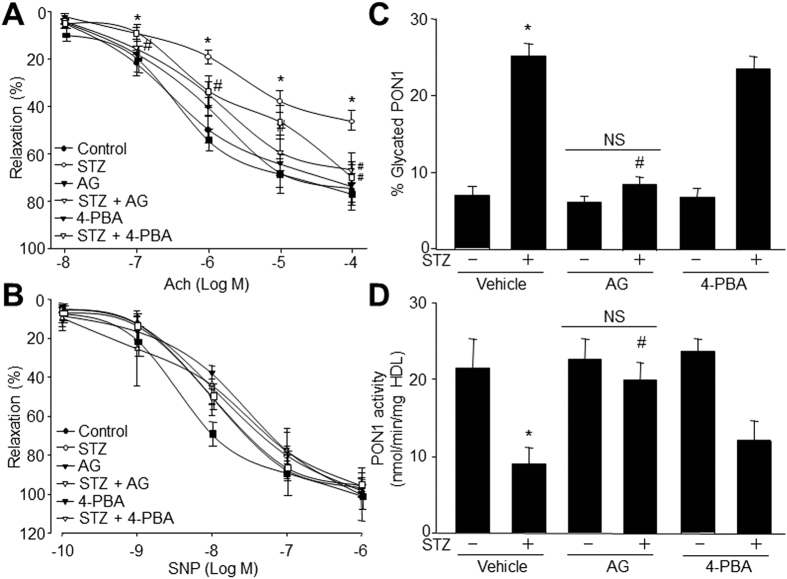
AGEs inhibitor amino guanidine and ER chaperone 4-PBA prevent endothelial dysfunctions in STZ-injected rats. SD rats were administrated with amino guanidine (100 mg/kg per day) or 4-PBA (1 g/kg/day) for 2 weeks prior to the induction of hyperglycemia for the following 4 weeks. Aortas from rats were cut into rings and were mounted in organ chamber to detect vessel bioactivity. (**A**) Endothelium-dependent relaxation of the aortic rings in response to Ach. (**B**) Endothelium-independent relaxation of the aortic rings in response to SNP. Each data point represents relaxation expressed as a percentage of the value obtained for PE-preconstricted aorta. Two aortic rings were isolated from each rat. (**C**) Serum levels of glycated PON1 in rats. (**D**) Serum PON1 activity in rats. 10–15 rats per group. *P < 0.05 *vs*. Control or Vehicle group. ^#^*P* < 0.05 *vs*. Diabetes.

Next, we assessed whether hyperglycemia via formation of glycated PON1 impairs endothelial function in diabetes. To this end, we treated rats with AGES inhibitor amino guanidine prior to the induction of persistent hyperglycemia by injecting STZ into rats. Endothelial function was determined by measuring Ach-induced endothelium-dependent relaxation. Treatment of diabetic rats with AG had no effects on the blood levels of glucose, cholesterol, and triglyceride (Table 1). As indicated in Fig. 4A, hyperglycemia impaired Ach-induced relaxation. Pretreatment with AG, which did not alter basal Ach-induced relaxation, reversed the reduction of Ach-induced vasorelaxation caused by hyperglycemia. In addition, the endothelium-independent vasorelaxation, as assayed by monitoring vasorelaxation to SNP, a NO donor, was unchanged among these groups (Fig. 4B), suggesting unchanged function of vascular smooth muscle cells in terms of nitric oxide. Overall, these results suggest that inhibition of protein glycation is sufficient to preserve endothelium-dependent relaxation impaired by hyperglycemia.

### AG abolishes PON1 glycation and reverses PON1 activity *in vivo*

To further test the role of PON1 glycation in response to hyperglycemia-induced vascular dysfunction, the serum levels of PON1 glycation and activity were assayed in rats. Similar to *in vitro* results in Fig. 1A–C, hyperglycemia increased serum levels of PON1 glycation and reduced serum PON1 activity (Fig. 4C and D). Importantly, administrations of AG reversed these abnormalities in STZ-induced hyperglycemic rats, indicating that hyperglycemia-induced endothelial dysfunction is related to PON1 glycation.

### Inhibition of ER stress by 4-PBA attenuates endothelial dysfunction in rats with hyperglycemia

Next, we assessed whether hyperglycemia via ER stress impairs endothelial function in diabetes. To this end, we treated rats with ER chaperone 4-PBA prior to the induction of persistent hyperglycemia. As indicated in Fig. 4A, pretreatment with 4-PBA, which did not alter basal Ach-induced relaxation, rescued Ach-induced vasorelaxation in rats with hyperglycemia, but had no effects on the blood levels of glucose, cholesterol, and triglyceride (Table 1). Also, 4-PBA did not alter the SNP-induced endothelium-independent vasorelaxation (Fig. 4B). Expectedly, 4-PBA had no effects on the serum levels of PON1 glycation and activity from hyperglycemic rats (Fig. 4C and D), indicating that 4-PBA functions as an ER stress inhibitor but not an inhibitor of AGEs.

### Gly-PON1 induces ER stress and endothelial dysfunction in rats

**Figure 5:**
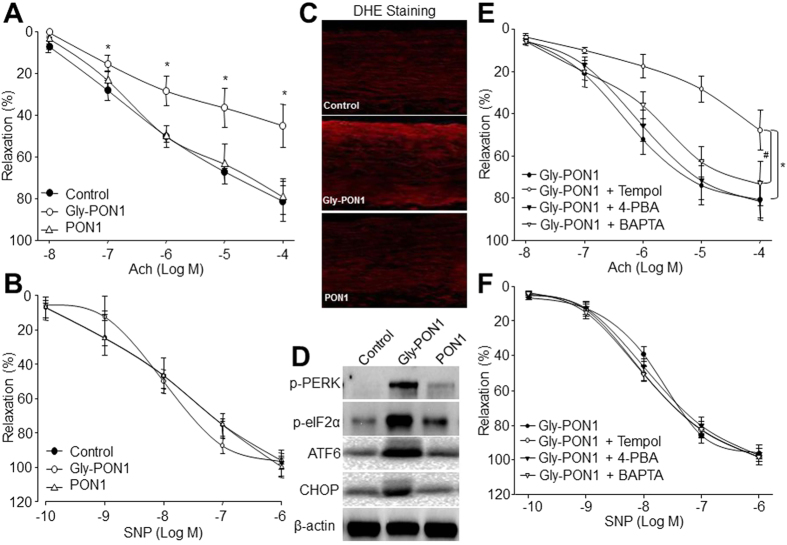
Glycated PON1 via ER stress induces endothelial dysfunction in rats. (**A**–**D**) SD rats were injected with native or glycated PON1 (1 mg/kg/day) for 7 consecutive days via tail vein. Aortas from rats were cut into rings and were mounted in organ chamber to detect vessel bioactivity. Endothelium-dependent relaxation of the aortic rings in response to Ach in (**A**), Endothelium-independent relaxation of the aortic rings in response to SNP in (**B**), ROS productions in (**C**), and the levels of p-PERK, p-eIF2α, ATF6 and CHOP in (**D**) were assayed. 10–15 rats in each group. *P <* *0.05 *vs*. Control rats. (**E**,**F**) SD rats were administrated with tempol (1 mM in the drinking water), 4-PBA (1 g/kg/day), and BAPTA (5 mg/kg per day) for 2 weeks followed by injection of glycated PON1 (1 mg/kg/day, 7 consecutive days) via tail vein. Aortas from rats were cut into rings and were mounted in organ chamber to detect vessel bioactivity. The relaxation was induced by Ach in (**D**) or SNP in (**E**). Each data point represents relaxation expressed as a percentage of the value obtained for PE-preconstricted aorta. Two aortic rings were isolated from each rat. 10–15 rats in each group. *P < 0.05 *vs* Gly-PON1 alone. The presented blots were cropped from the full blots shown in Supplementary Figure S11.

The role of PON1 glycation in diabetic endothelial dysfunction was further confirmed by injecting gly-PON1 into rats. As indicated in Table 2, both native PON1 and gly-PON1 did not alter the serum levels of glucose, cholesterol, and triglyceride. Compared to control rats, native PON1 did not affect vascular functions. Importantly, injection of gly-PON1, which mimicked the effects of hyperglycemia on endothelial function (Fig. 4A), inhibited Ach-induced endothelium-dependent relaxation, but had no effects on SNP-induced endothelium-independent relaxation (Fig. 5A and B). Further, DHE analysis indicated that gly-PON1, rather than native PON1, increased superoxide productions in arterial walls from rats (Fig. 5C), as well as increased ER stress (Fig. 5D). Taking these data together, it demonstrates that PON1 glycation is an inducer of ER stress and a risk factor of endothelial dysfunction in diabetes.

### Tempol, BAPTA, 4-PBA prevent endothelial dysfunction in rats injected with glycated PON1

Knowing the key role of the superoxide/SERCA oxidation/Ca^2+^ axis in gly-PON1-induced ER stress *in vitro*, we finally determined whether suppression of this axis prevented gly-PON1-induced endothelial dysfunction *in vivo*. To this point, SD rats were chronically administrated with tempol, BAPTA, and ER chaperone 4-PBA followed by gly-PON1 injection. As shown in Fig. 5E and F, tempol, BAPTA, and 4-PBA protected Ach-induced endothelium-dependent relaxation but had no effects on SNP-induced endothelium-independent relaxation. Besides, tempol, BAPTA, and 4-PBA had not affect the levels of blood glucose, cholesterol, and triglyceride (Table 2). These data further provide the evidence that gly-PON1 impairs endothelial function via the activation of superoxide/SERCA oxidation/Ca^2+^ axis in diabetes.

## Discussion

In this study, we have demonstrated that PON1 glycation causes aberrant ER stress via the oxidation of SERCA in endothelial cells. We have also provided evidence that activation of the superoxide/SERCA oxidation/Ca^2+^ axis mediates gly-PON1-enhanced SERCA oxidation and consequent ER stress *in vitro* and *in vivo*. Finally, we found chronic administration of tempol, BAPTA, and 4-PBA restored endothelial function impaired by gly-PON1.

The major discovery of this present study is that we identified glycation of PON1 as a risk factor of endothelial dysfunction in diabetes. It has previously been reported that glycation inhibits PON1 activity towards paraoxon in type 2 diabetes^36^. In this study, we consistently found that glycation of PON1 not only inhibited paraoxon hydrolysis but also induced endothelial dysfunction in diabetic rats. It is therefore possible that the increased *in vivo* glycation of PON1 leads to its glyoxidation and reduction of activity, and is responsible for the derangement of membrane hydroperoxide metabolism found in HDL from people with diabetes. Although inhibition of PON1 by glycation by AG appears to be the most likely cause of our results, we cannot discount the possibility that glycation of other HDL proteins affects PON1 activity and/or the ability of HDL to metabolize hydroperoxides. This could be by disrupting HDL structure, or glycation of apoA1 could affect its ability to modulate PON1 function.

Mechanically, we demonstrated that gly-PON1 increases ER stress by superoxide-dependent oxidation and inhibition of SERCA, a key enzyme that controls intracellular Ca^2+^. Further, gly-PON1 increases the oxidation of Cys674, a known site for SERCA glutathiolation and oxidation, likely via NAD(P)H oxidase-derived superoxide. Our observations are in line with several published studies that have demonstrated that SERCA is prone to oxidation at one of its thiol groups under certain conditions and that its oxidation is significantly increased in atherosclerosis and diabetes^28^,^37^,^38^. Importantly, we found that chronic administration of tempol significantly inhibited ER stress and normalized endothelial dysfunction. Thus, superoxide might be a common ER stress inducer in different cell types including endothelial cells in diabetes. Interestingly, ER stress inducer such as tunicamycin or thapsigargin induces Orai1 and STIM1 expression in multiple cells and high glucose is related to an increase in Orai1 and STIM1 activity^39^,^40^. In this present study, we did not see any difference in the expression of Orai1 and STIM1. We propose that this discrepancy may be explained by the difference of cell types, such as HUVECs and gly-PON1 used in this study.

In summary, this study identifies glycated PON1 as a novel risk factor to diabetic vascular complications and uncovers the mechanism by how gly-PON1 induces endothelial dysfunction via ER stress, which is mediated by superoxide/SERCA oxidation/Ca^2+^ axis in diabetes. Therefore, inhibition of oxidative stress by tempol, chelating intracellular Ca^2+^ by BAPTA, and ER chaperone 4-PBA would be considered to prevent endothelial dysfunction and reduce vascular complications.

## Additional Information

**How to cite this article:** Yu, W. *et al*. Glycation of paraoxonase 1 by high glucose instigates endoplasmic reticulum stress to induce endothelial dysfunction *in vivo. Sci. Rep.* **7**, 45827; doi: 10.1038/srep45827 (2017).

**Publisher's note:** Springer Nature remains neutral with regard to jurisdictional claims in published maps and institutional affiliations.

## Supplementary Material

Supplementary Data

## Figures and Tables

**Table 1:** Serum lipid and glucose levels in rats injected with STZ.

Groups	N	Glucose (mg/dl)	Cholesterol (mM)	Triglyceride (mM)
Control	10	152 ± 27	1.70 ± 0.62	0.70 ± 0.24
STZ	13	378 ± 39*	1.72 ± 0.50	0.78 ± 0.19
AG	15	164 ± 19	1.68 ± 0.59	0.69 ± 0.13
STZ + AG	13	365 ± 46*	1.73 ± 0.81	0.77 ± 0.11
4-PBA	12	158 ± 21	1.78 ± 0.52	0.74 ± 0.23
STZ + 4-PBA	11	359 ± 36*	1.69 ± 0.61	0.70 ± 0.21

Note: SD rats were administrated with amino guanidine (100 mg/kg per day) or 4-PBA (1 g/kg/day) for 2 weeks prior to the induction of hyperglycemia for the following 4 weeks. Four weeks after STZ injection, the rats in each group were anesthetized with sodium pentabarbitone (30 mg/kg, I.P.) and exsanguinated. Blood was collected to measure serum levels of lipid and glucose. *P < 0.05 *VS* Control rats.

**Table 2:** Serum lipid and glucose levels in rats injected with gly-PON1.

Groups	N	Cholesterol (mM)	Triglyceride (mM)	Glucose (mg/dl)
Control	13	11.53 ± 2.18	0.89 ± 0.17	158 ± 40
PON1	11	12.07 ± 5.13	0.97 ± 0.26	162 ± 27
Gly-PON1	14	13.74 ± 3.97	0.86 ± 0.14	159 ± 21
Gly-PON1 + Tempol	15	11.88 ± 1.96	0.81 ± 0.18	166 ± 12
Gly-PON1 + BAPTA	10	13.35 ± 2.38	0.83 ± 0.10	174 ± 33
Gly-PON1 + 4-PBA	12	12.99 ± 3.72	0.92 ± 0.17	177 ± 36

Note: SD rats were administrated with tempol (1 mM in the drinking water), 4-PBA (1 g/kg/day), and BAPTA (5 mg/kg per day) for 2 weeks followed by injection of glycated PON1 (1 mg/kg/day, 7 consecutive days) via tail vein. At the end of experiments, blood was collected to measure serum levels of lipid and glucose.
