# Supplementary material for: Glycation of paraoxonase 1 by high glucose instigates endoplasmic reticulum stress to induce endothelial dysfunction in vivo
Source: Sci Rep. 2017 Apr 4;7:45827. doi: 10.1038/srep45827 (PMC5379182; doi:10.1038/srep45827)
Supplement: Supplementary Data [file srep45827-s1.pdf]

Supplementary Information for

Glycation of paraoxonase 1 by high glucose instigates endoplasmic reticulum stress to induce endothelial dysfunction *in vivo*

Wei Yu[1,2,#], Xiaoli Liu[2,#], Liru Feng[2], Hui Yang[2], Weiye Yu[2], Tiejian Feng[2], Shuangxi Wang[3], Jun Wang[2,*], Ning Liu[1,*]

[1]Central Laboratory, Second Hospital, Jilin University, Changchun 130041, China; [2]Shenzhen Center for Chronic Disease Control, Shenzhen 518020, China; [3]Department of Pharmacology, College of Pharmacy, Xinxiang Medical University, Xinxiang, 453003, China

[#] These authors contributed equally to this work.

* Correspondence to Jun Wang or Ning Liu, Email junwangwh@hotmail.com or liu_ning@jlu.edu.cn

Running Title: PON1 glycation induces ER stress

Contents

1. Supplementary Figure Legends S1-S11
2. Supplementary Figure S1-S11

Supplementary Figure Legends

Supplementary Figure S1. Full western blot pictures shown in Figure 1A.

Supplementary Figure S2. Full blots for blots shown in Figure 1D.

Supplementary Figure S3. Full blots for blots shown in Figure 1E.

Supplementary Figure S4. Full blots for blots shown in Figure 2A.

Supplementary Figure S5. Full blots for blots shown in Figure 2D.

Supplementary Figure S6. Full blots for blots shown in Figure 2E.

Supplementary Figure S7. Full blots for blots shown in Figure 2G.

Supplementary Figure S8. Full blots for blots shown in Figure 2G.

Supplementary Figure S9. Full blots for blots shown in Figure 3B.

Supplementary Figure S10. Full blots for blots shown in Figure 3C.

Supplementary Figure S11. Full blots for blots shown in Figure 5D.

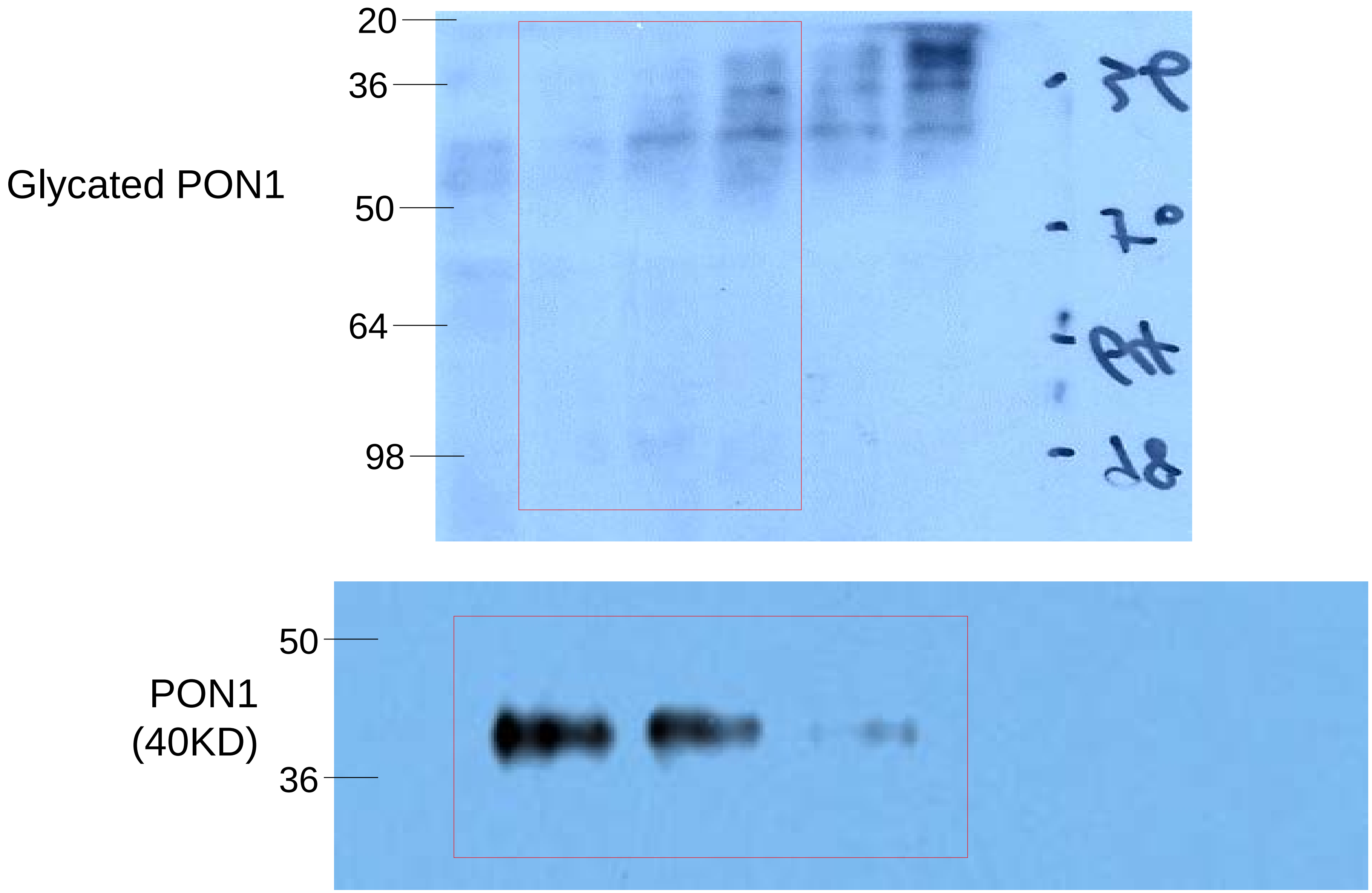

Supplementary Figure S1

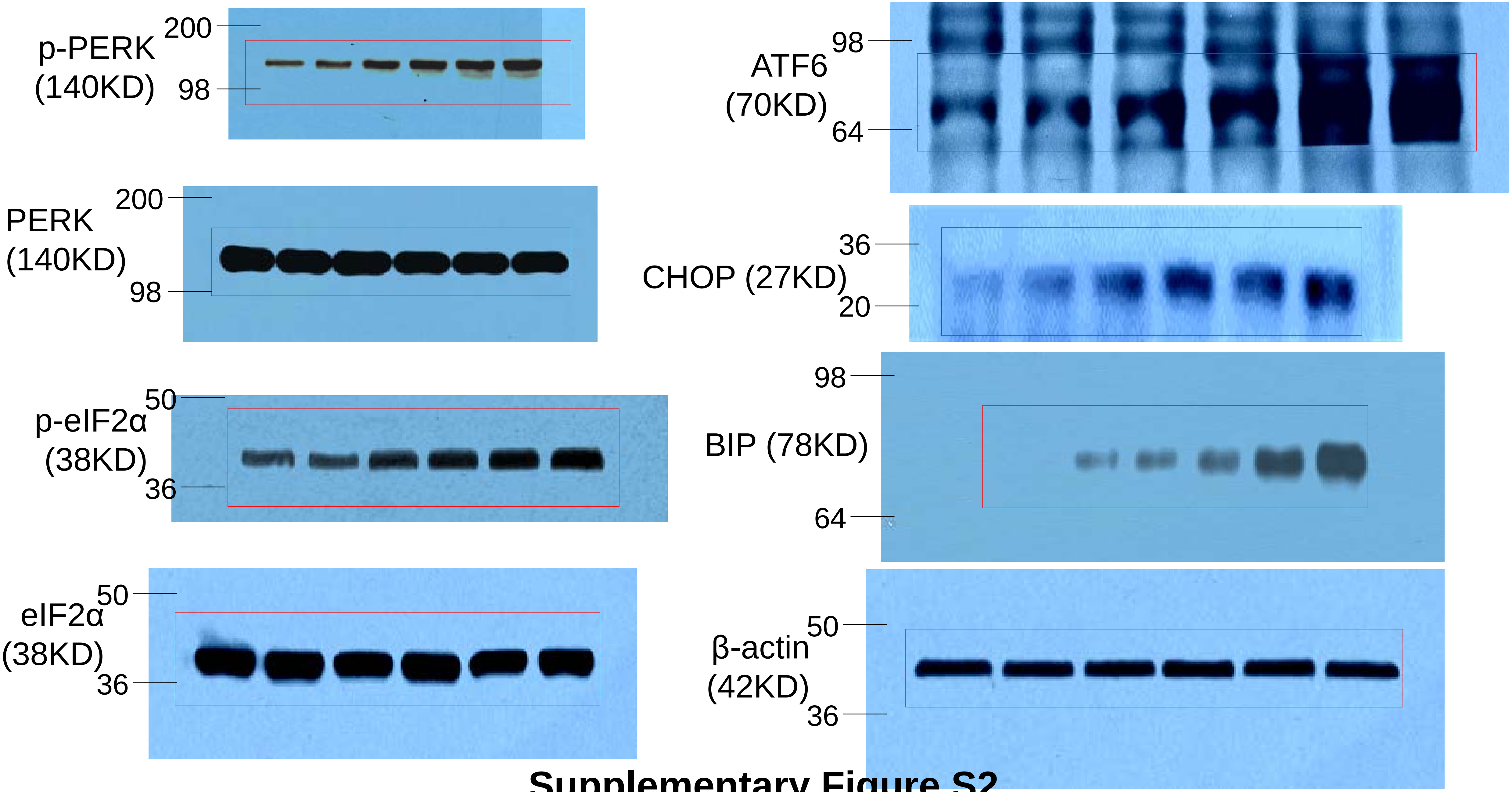

Supplementary Figure S2

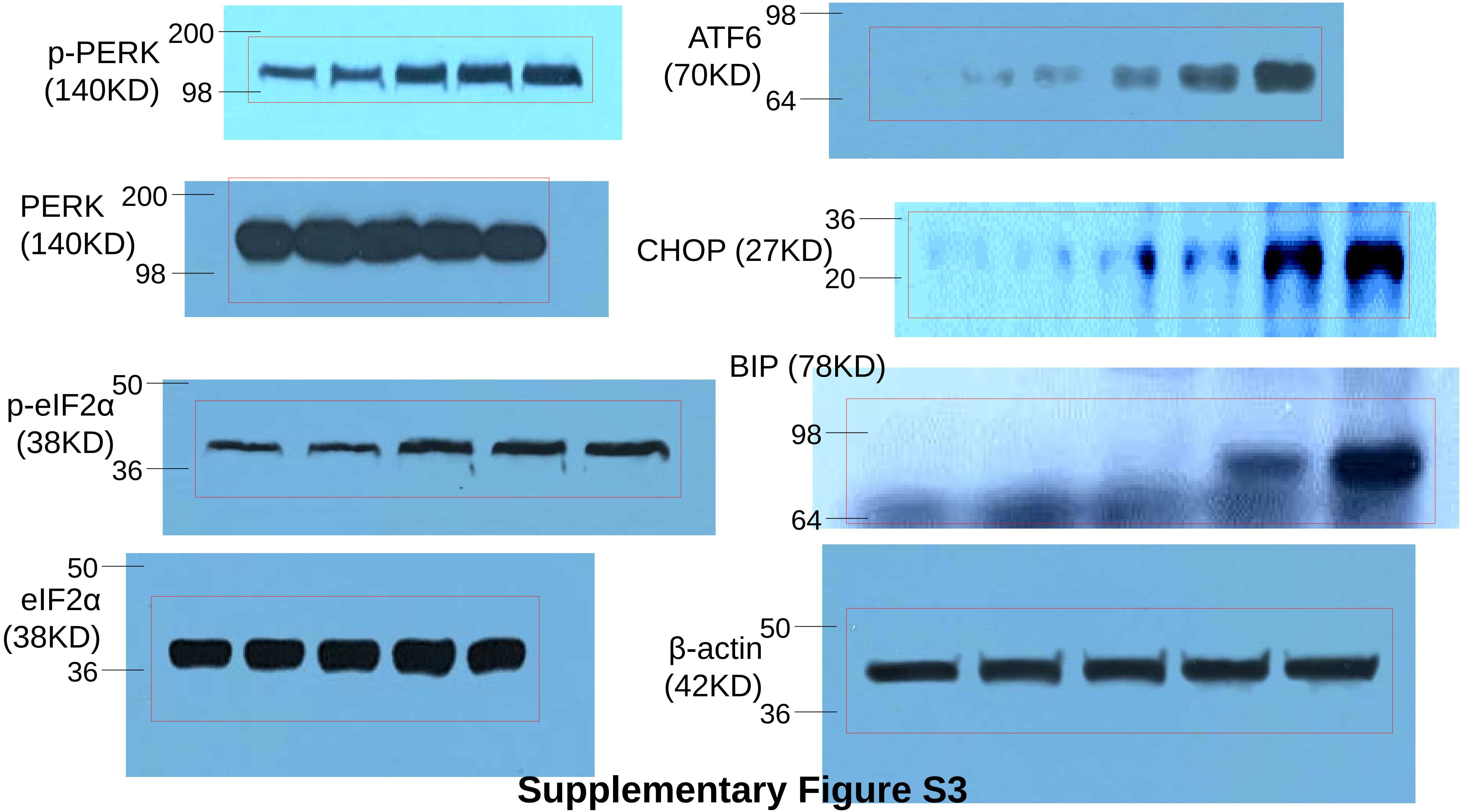

Supplementary Figure S3

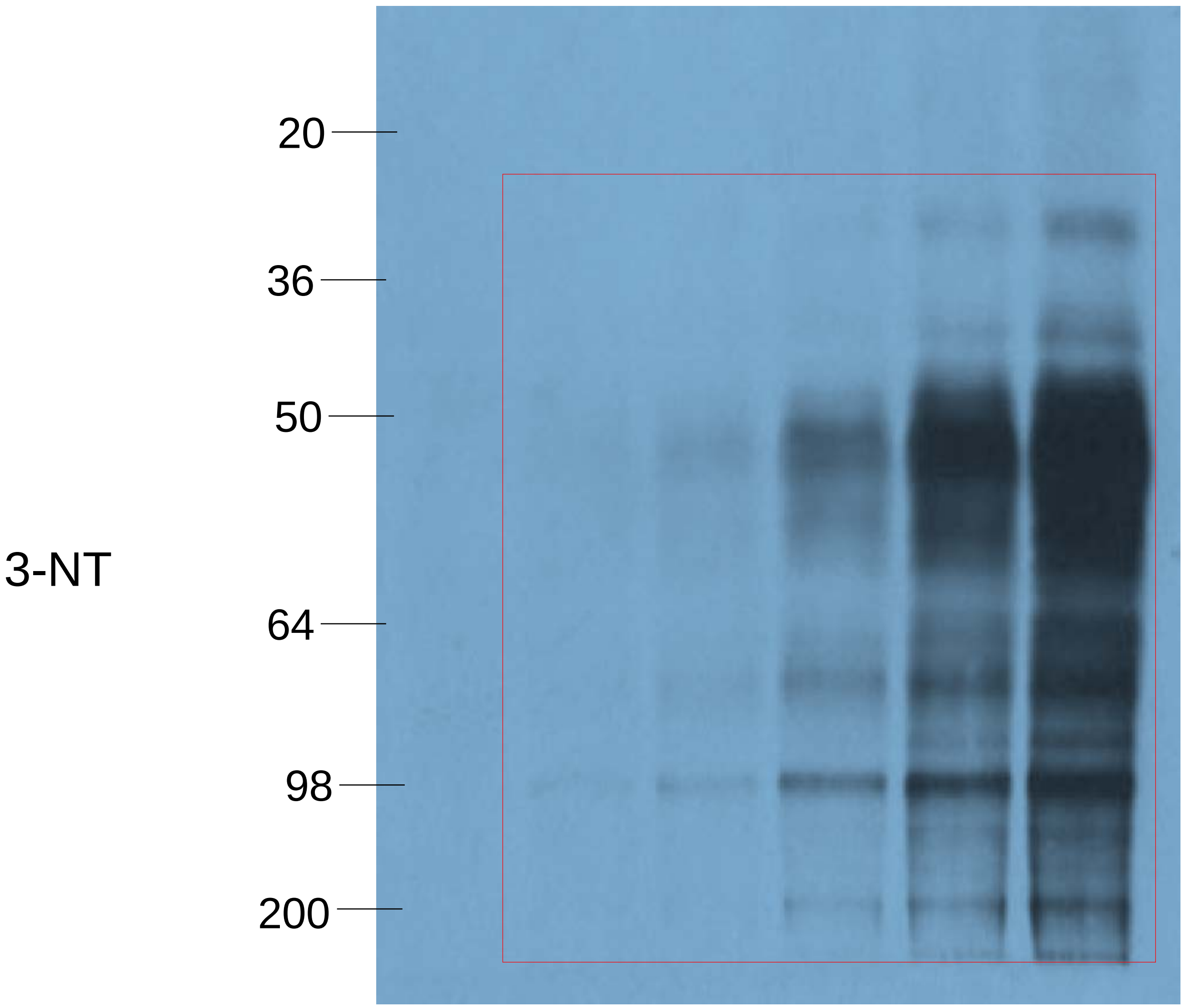

Supplementary Figure S4

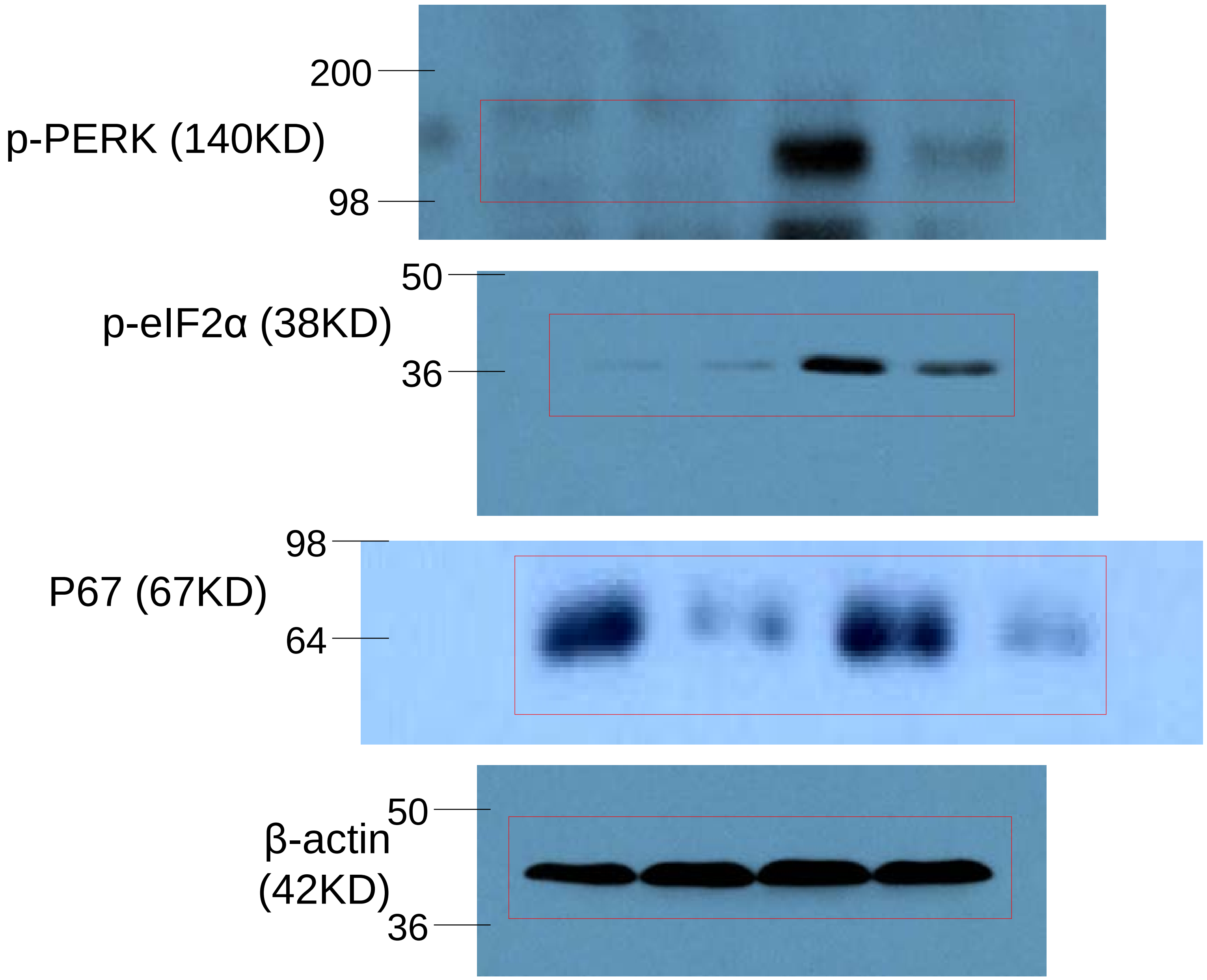

Supplementary Figure S5

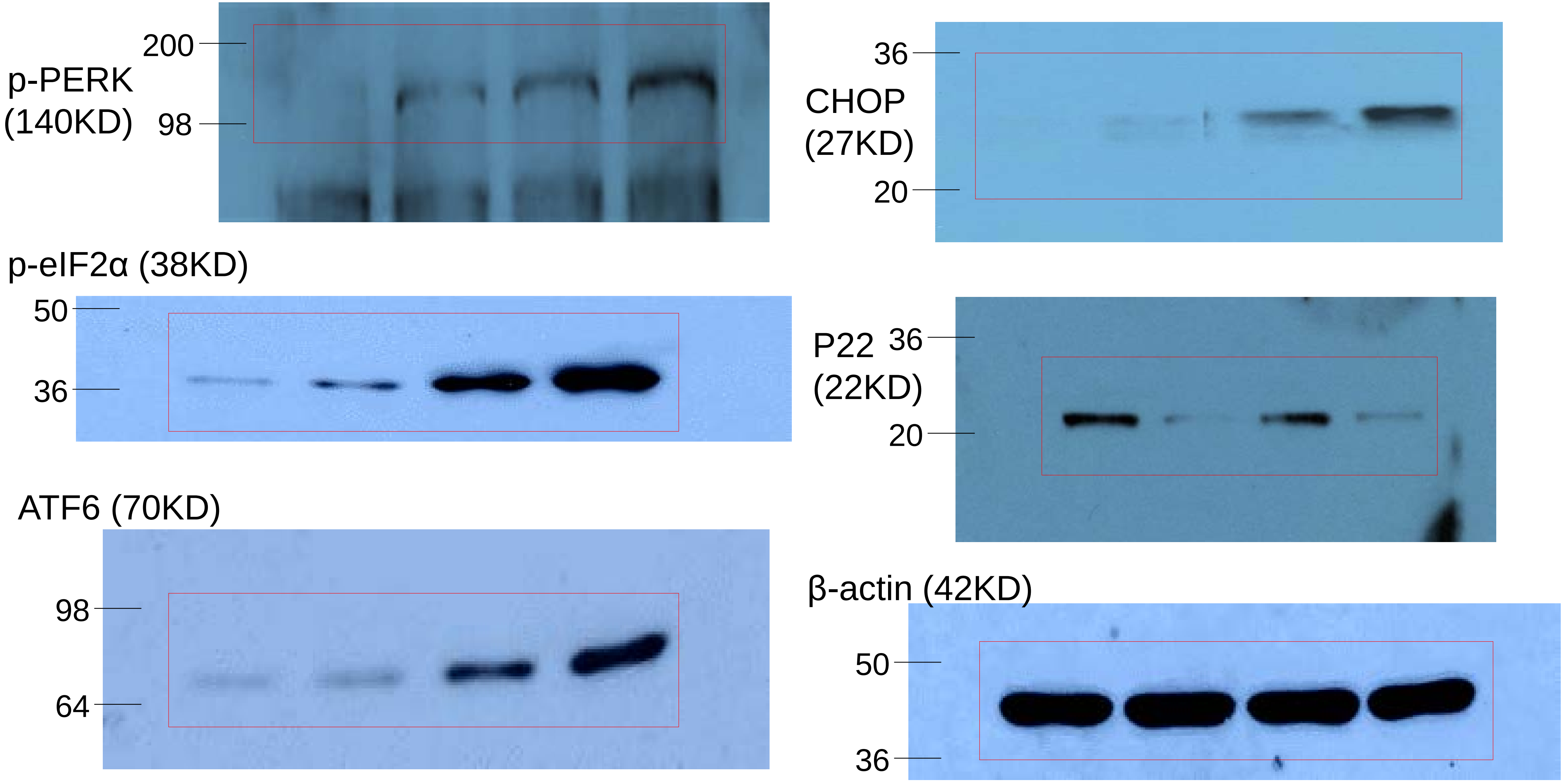

Supplementary Figure S6

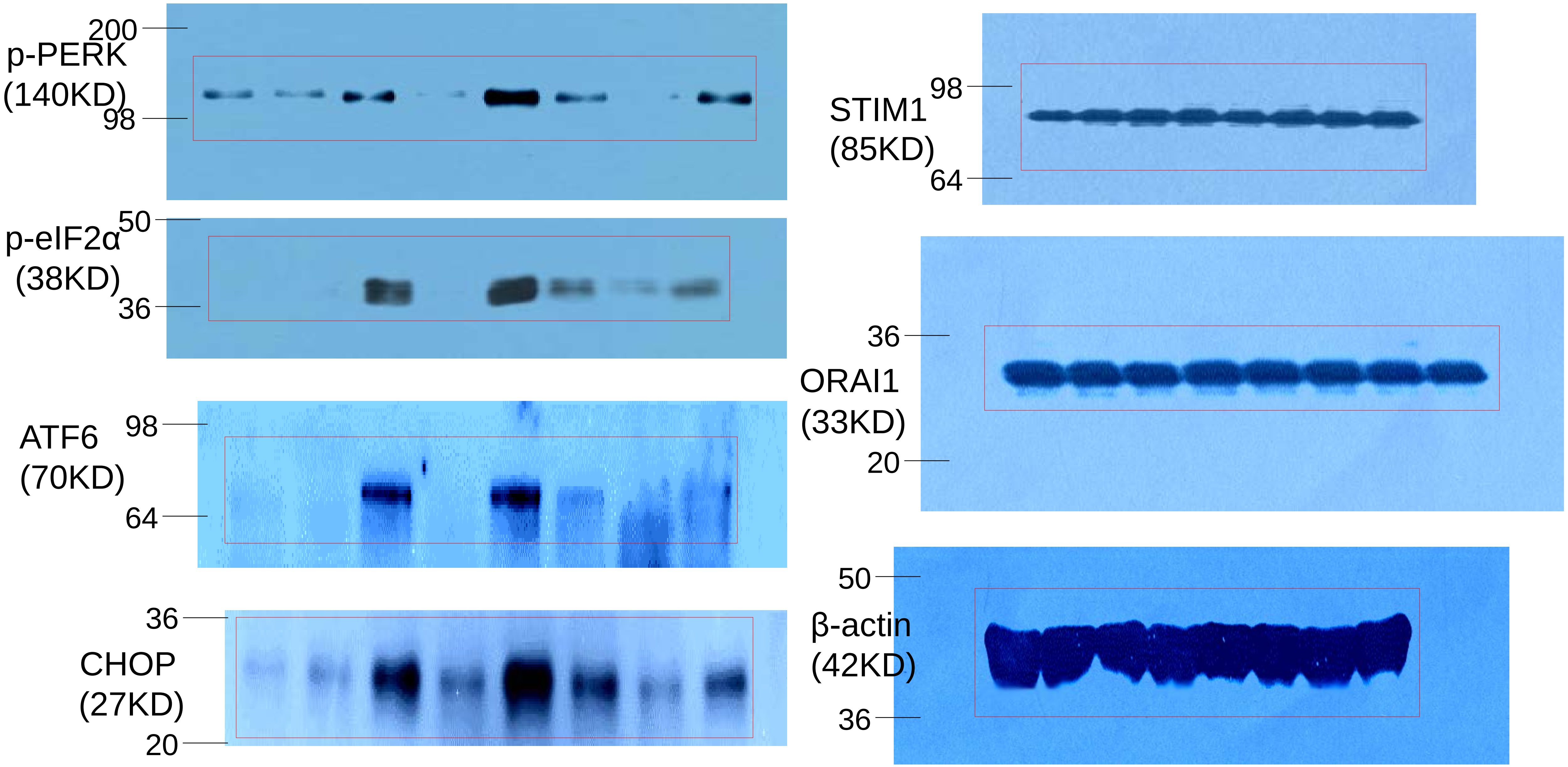

Supplementary Figure S7

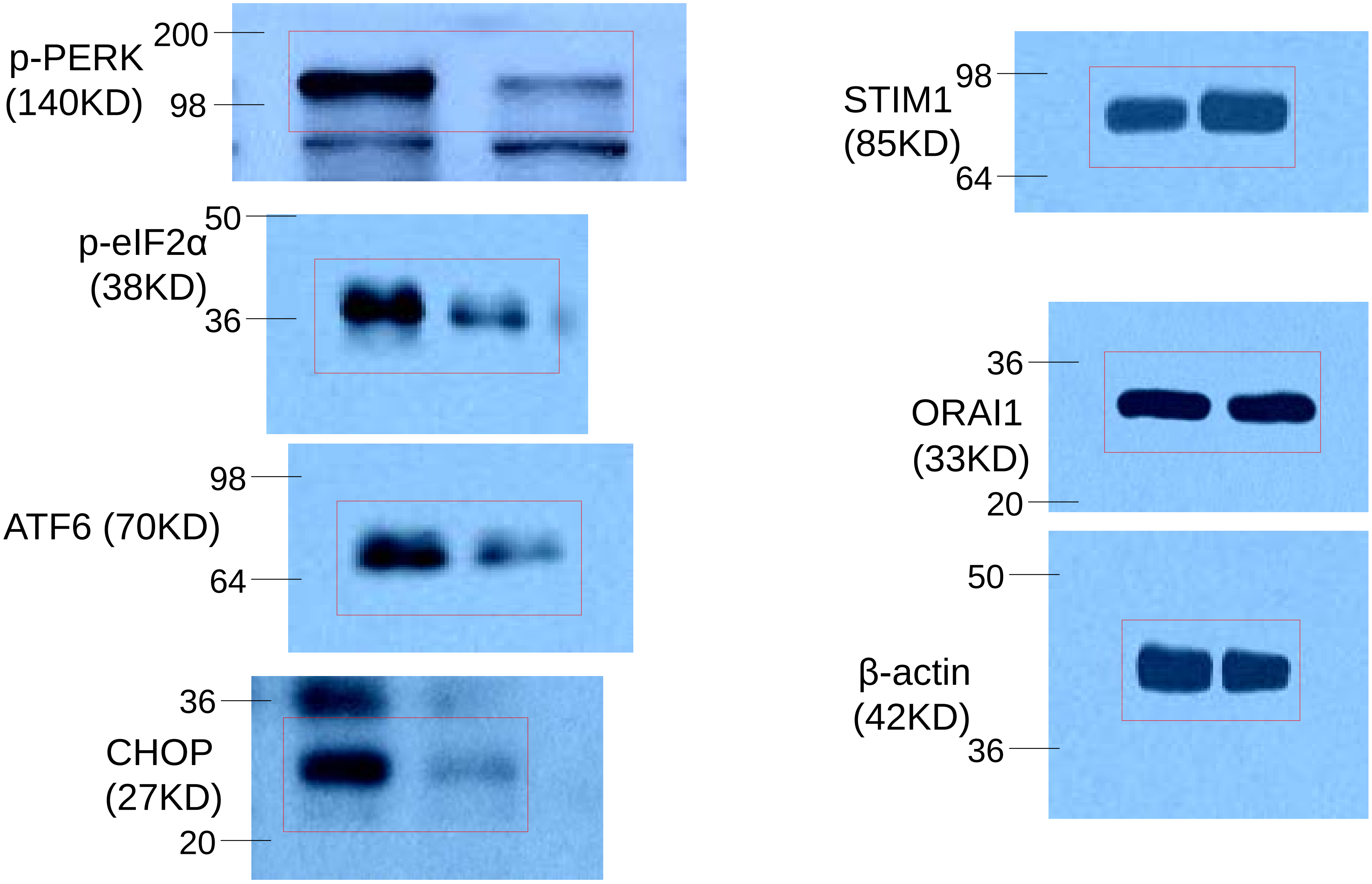

Supplementary Figure S8

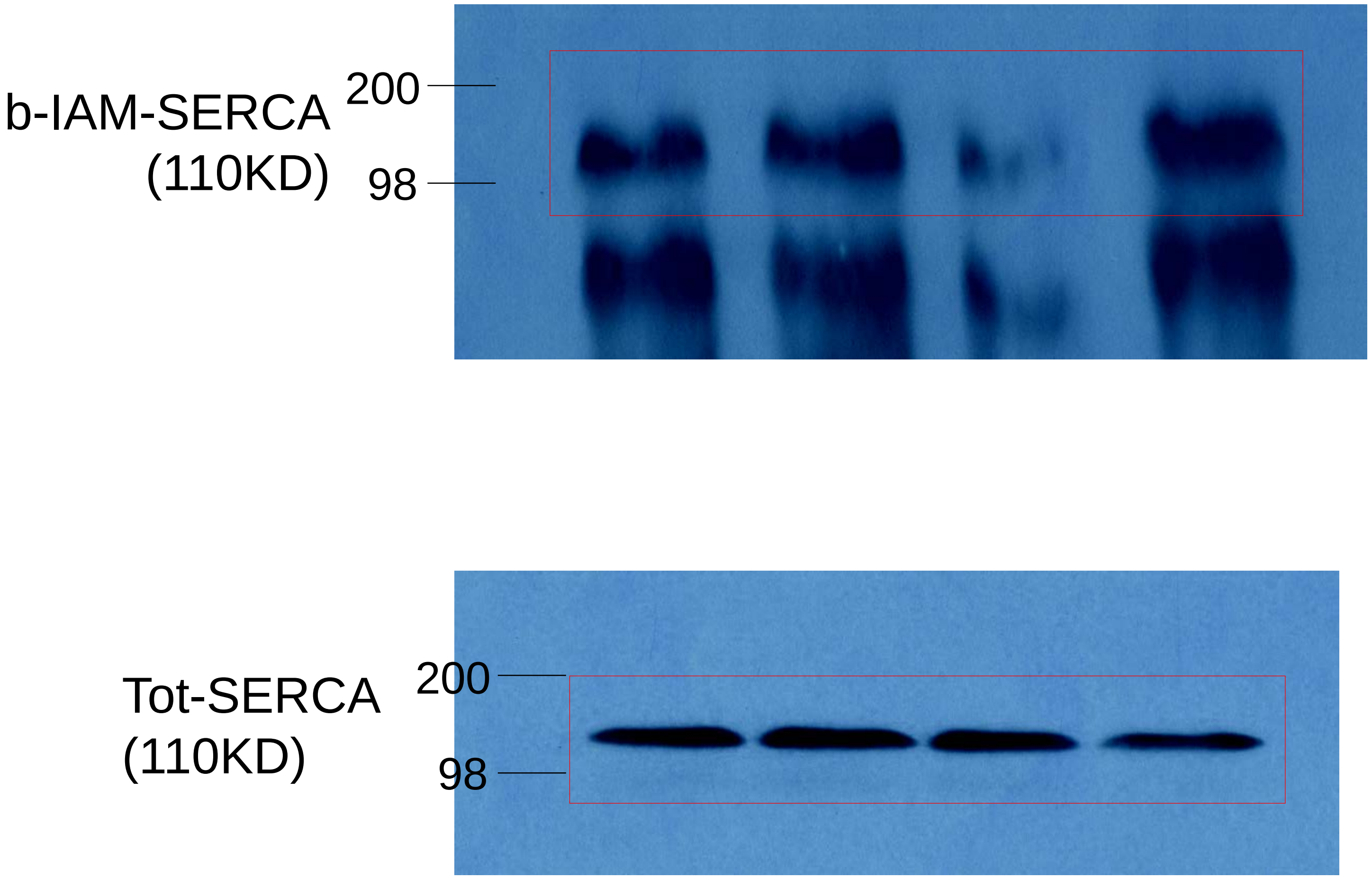

Supplementary Figure S9

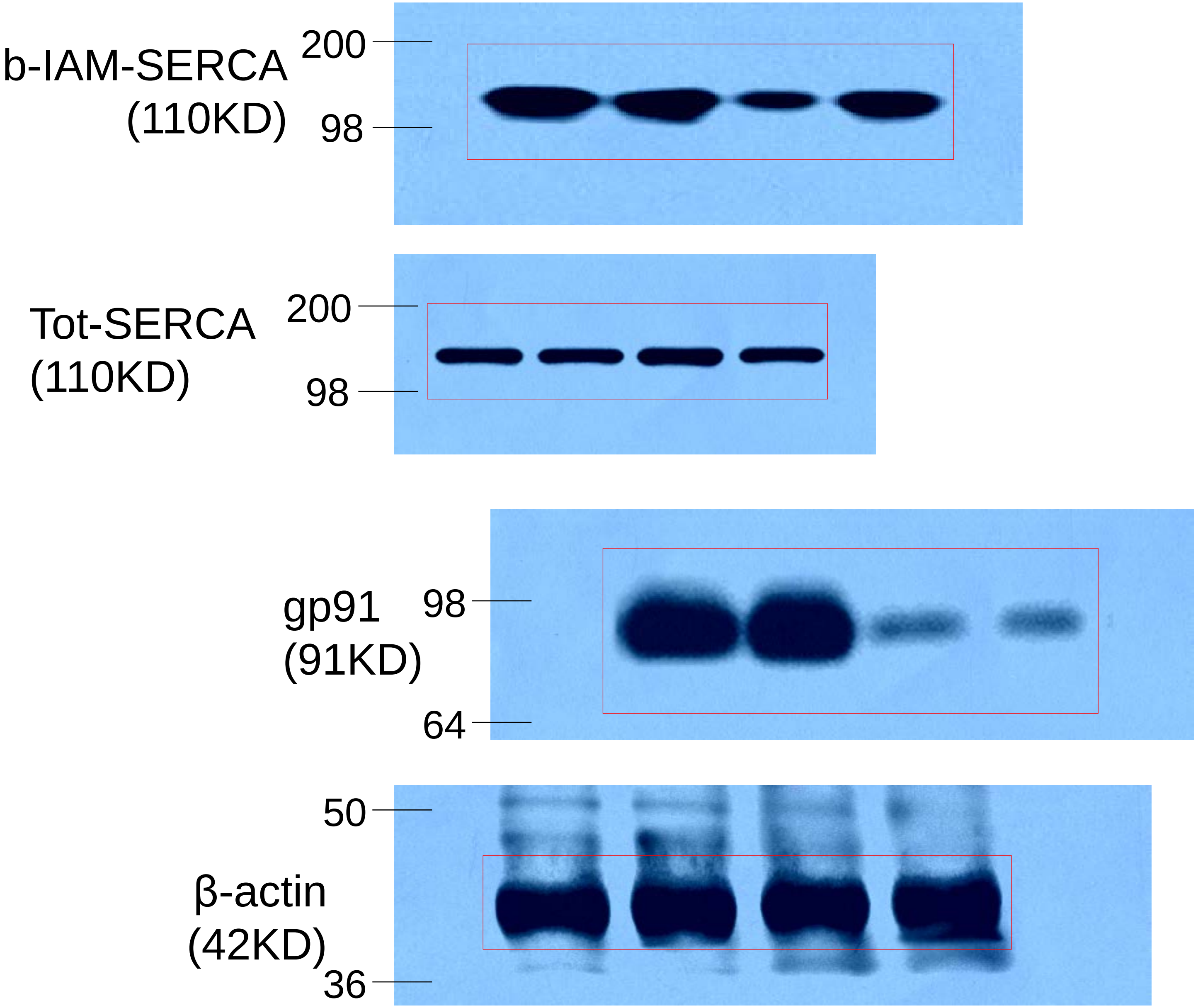

Supplementary Figure S10

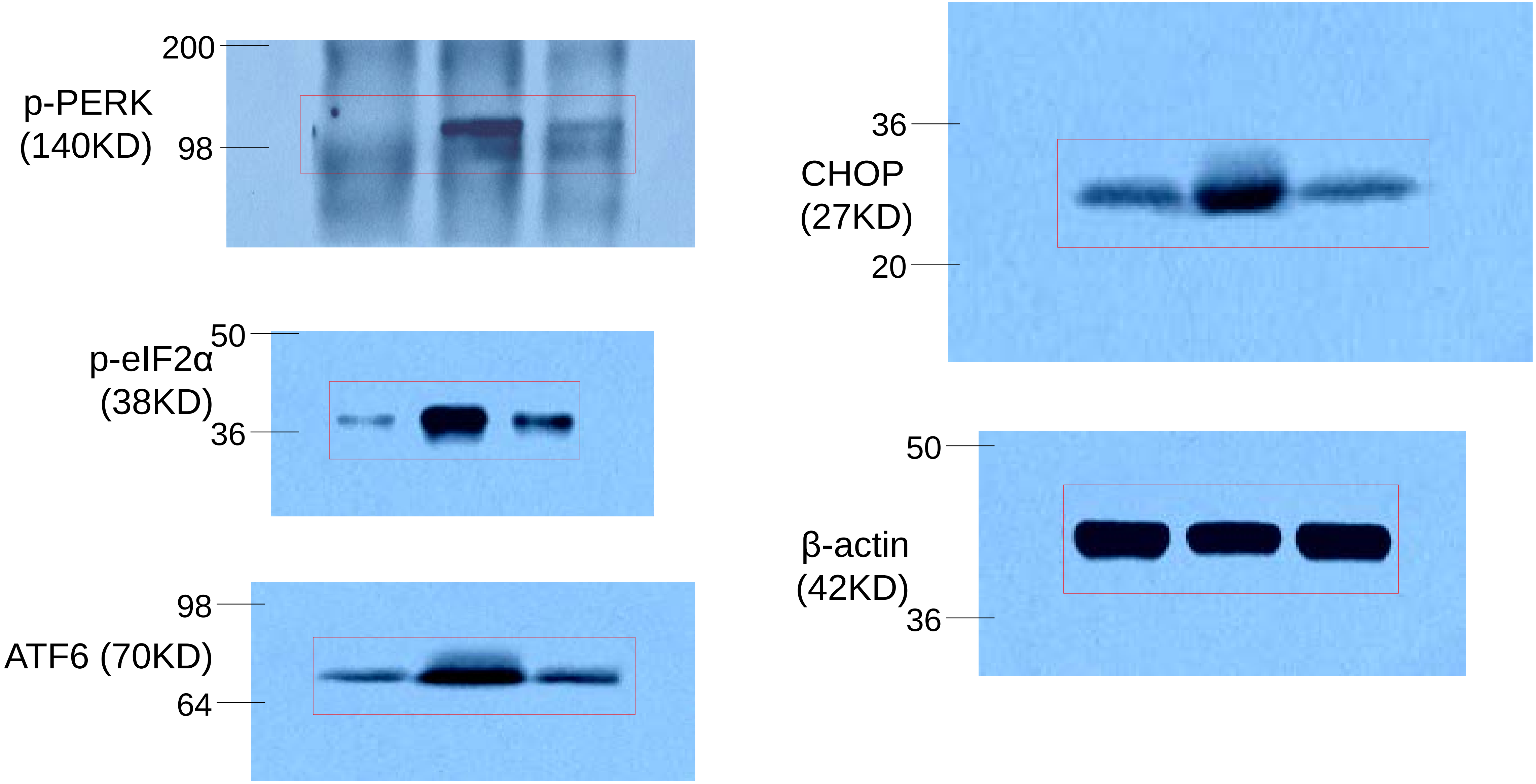

Supplementary Figure S11
